# Micron-scale human enamel layer characterization after orthodontic bracket debonding by intensity-based layer segmentation in optical coherence tomography images

**DOI:** 10.1038/s41598-021-90354-9

**Published:** 2021-05-25

**Authors:** Naresh Kumar Ravichandran, Hemanth Tumkur Lakshmikantha, Hyo-Sang Park, Mansik Jeon, Jeehyun Kim

**Affiliations:** 1 School of Electronic and Electrical Engineering, Kyungpook National University, 80 Daehakro, Bukgu, Daegu, 41566 Republic of Korea; 2 Center for Scientific Instrumentation, Korea Basic Science Institute, 169148 Gwahakro Yuseonggu, Daejeon, 34133 Republic of Korea; 3 Department of Orthodontics, School of Dentistry, Kyungpook National University, Daegu, 41940 Republic of Korea

**Keywords:** Dental diseases, Biophotonics, Near-infrared spectroscopy, Imaging and sensing

## Abstract

In clinical orthodontic practice, fixed brackets are widely used for tooth movement and adjustments. Although years of research and development have improved the workability of fixed orthodontic brackets, there are still controversies regarding its plausible destructive influence on the enamel surface of tooth. This, in turn, makes the quantitative assessment of the enamel surface after specific orthodontic treatment procedures important in order to opt for the most effective treatment procedure. Through this study, we show the practical applicability of optical coherence tomography (OCT) as a non-ionizing and nondestructive assessment tool for measuring enamel loss after each step of orthodontic bracket bonding. Two-dimensional and volumetric OCT images are used for the evaluation of the tooth enamel. From the depth intensity profile analysis of cross-sectional OCT images, the changes in the individual internal layer thickness are calculated. A software algorithm was developed to evaluate the structural connectivity in the enamel for analyzing enamel loss on the tooth surface and for detecting enamel abrasion. An intensity-based layer segmentation algorithm is also developed to analyze and evaluate enamel wear in the tooth after each step. Using the proposed algorithms, the total enamel present after each treatment procedure was measured and tabulated for analysis.

## Introduction

The tissue that covers the dentin in a tooth is called the enamel. Usually, the thickness of the enamel ranges from 1000 *µ*m to 1500 *µ*m. About 98% of the enamel is composed of an inorganic substance called dihydroxyapatite crystal^1,2^. Most orthodontic treatments primarily involve the bonding of brackets to the enamel surface. Different types of bracket attachment methods are currently practiced. The most essential concern during orthodontic treatment is the state of the enamel before and after the treatment process. Numerous methods are practiced for the removal of adhesive/bonding agent residues in orthodontics, and efficient methods for the removal of residue play a vital role in the resultant enamel integrity^3^. For instance, there are techniques of adhesive attachment to the enamel surface that enable the rapid development of orthodontic treatments with bonded braces^4–6^. These methods are continuously adapted and new approaches are proposed for effective and efficient adhesive application techniques to aid orthodontic treatment without damaging the tooth surface or bracket integrity. Controversially, when it comes to fixed orthodontic brackets and their supporting structures, the potential damage to the tooth surface has always been a topic of scrutiny^7,8^. From previous research, it is evident that enamel breakouts after debonding are common in about 27% of all cases. In^8^, this is also observed for the use of uncoated and pre-coated brackets. This highlights the need for quantitative and qualitative assessments of tooth enamel.

Conventional dental imaging and evaluation techniques such as visual inspection^9^, X-ray computed tomography^10^, scanning electron microscopy^11^, atomic force microscopy^12^, micro-computed tomography^13^, cone-beam computed tomography^14^, and dental radiography^15^ have been widely used to assess enamel structure and its integrity. These techniques need either sectioning processes for deep hard tissue assessments or low-level radiation for imaging of the enamel and dentin. These limitations can be overcome by utilizing optical coherence tomography (OCT) for non-ionizing and nondestructive evaluation of biological specimens. OCT has been explored as a substitute for nondestructive diagnosis of biological specimens in the field of ophthalmology^16–18^, deep brain imaging^19,20^, and endoscopy^21^. Furthermore, OCT been used for studies related to disease progression in agronomy^22–24^, entomology^25,26^, and in industrial defect inspections^27,28^. In the field of dentistry and oral health diagnosis, OCT has consistently proven to be a reliable imaging technique for diagnosis and research^1,29–31^. Furthermore, OCT has consistently seen a steady rise as a reliable imaging modality in the field of demntal related experimental and clinical applications like diagnostics imaging tool^32^, to evaluate the tooth deacay rate^33^, enamel morphology assessment and bracket bond strength estimation^34^, and for enamel surface evaluation post etching process by Er,Cr:YSGG laser with varied repetition rate and laser power as paramaters^35^.

One of the primary challenges of orthodontic treatment is the evaluation and assessment of enamel structures at different time intervals and different stages of treatment (diagnosis)^36–38^. This is attributable to the difficulties experienced during the automatic segmentation of the enamel structure from its surrounding tissues. The use of OCT for enamel thickness measurement and evaluation of bracket bonding strength is presented in ^39^, along with the use of a dental paraphernalia and an OCT handpiece. This necessitates the development of an automated detection algorithm for enamel segmentation in OCT images that can be used to assess structural changes during different orthodontic treatment procedures^40,41^. OCT enables the non-invasive internal structural visualization of maximum enamel thickness and the desired tooth surface.

A dedicated auto detection algorithm for enamel by the processing of OCT images will enable a more straightforward quantification of the structural changes that occur during treatment procedures. Furthermore, by quantifying the efficiency of tooth cleaning after bracket bonding and bracket removal and the residue cleaning process, researchers may be able to quantify and evaluate the optimal treatment protocol for orthodontic treatment.

## Materials and methods

### Sample armamentarium

All the teeth samples used in this study were obtained from the department of orthodontics, school of dentistry, Kyungpook National University, Daegu, Republic of Korea. All the experiments were approved and was carried out as per the guidelines of the Institutional Animal and Human Care and Use Committee of Kyungpook National University (No. 2017-0145-1). The collected tooth samples, which were extracted for orthodontic and periodontal reasons, were all premolars that belonged to patients within the age group of 20-30 years. The collected samples were examined individually, and the final set of samples were selected based on uniformity. After the teeth samples were selected, they were debrided (to remove any residual soft tissues) and stored (anonymously, without any retraceable or identifiable information of patients) in a solution mixture of demineralized water and crystal thymol (0.1%) at room temperature 25 °C; the samples were used in experiments within two weeks from the day of extraction. The cleaning step, with the help of the abrasive material, is avoided to prevent any unintentional damage to the sample surface. The surfaces of the teeth specimens are cleaned with a fluoride-free toothpaste with gentle pressure. Following this, the samples were rinsed gently with distilled water and dried by compressed air.

A total of 30 teeth were used in this study. The specimens were mounted on a wax brick to maintain stability of the samples. The treatment procedures were carried on the mounted teeth samples. All teeth samples were imaged using the OCT system prior to treatment for the purpose of establishing a control group. Thereafter, all specimens underwent the process of preparation for bracket bonding to the tooth surface. The procedures involved first treating all the specimens prophylactically using plain pumice for 10 seconds on the vestibular surfaces, then rinsing with distilled water and drying with compressed air for 15 seconds. The next step is to etch the surface of the tooth. Etching of tooth surface is performed using 37% orthophosphoric acid Blue Etch (CERKAMED, Poland) for about 15 seconds, followed by rinsing with distilled water and drying by compressed air for 15 seconds.

The adhesive compound Orthosolo (ORMCO, USA) was applied to the etched surface. Both the etch and the adhesive compound were applied using an applicator tip, which is commonly used in restorative dentistry to apply the chemicals to the selected areas of the specimens. Then, a bracket was attached to each tooth surface using clamping tweezers so that the center of the bracket was placed at a distance of 3.5 mm below the edge of the occlusal surface (i.e., in the middle section of the mesial-distal axis of the tooth) and pressed firmly in place. The excess resin was removed from the periphery of the bracket base using a dental explorer, followed by light curing for 15 seconds with a halogen unit (Demetron A2; DEMETRON, Switzerland). As reported by Eliades *et al*.^42^, and to ensure maximum bond strength, the brackets were removed (debond) 7 days after bonding by gently squeezing the mesial and distal wings with bracket removal pliers (ORMCO, Glendora, California, USA).

The adhesive remnants were removed with caution without inducing any damage to the tooth surfaces using a 12-blade tungsten carbide bur (H247; KOMET, Germany) driven on a low speed handpiece at 20,000 rpm. The complete removal of the resin was confirmed by visual inspection. Finally, to reattach a new bracket on the tooth surface, the aforementioned procedures were repeated.

### OCT system setup

**Figure 1:**
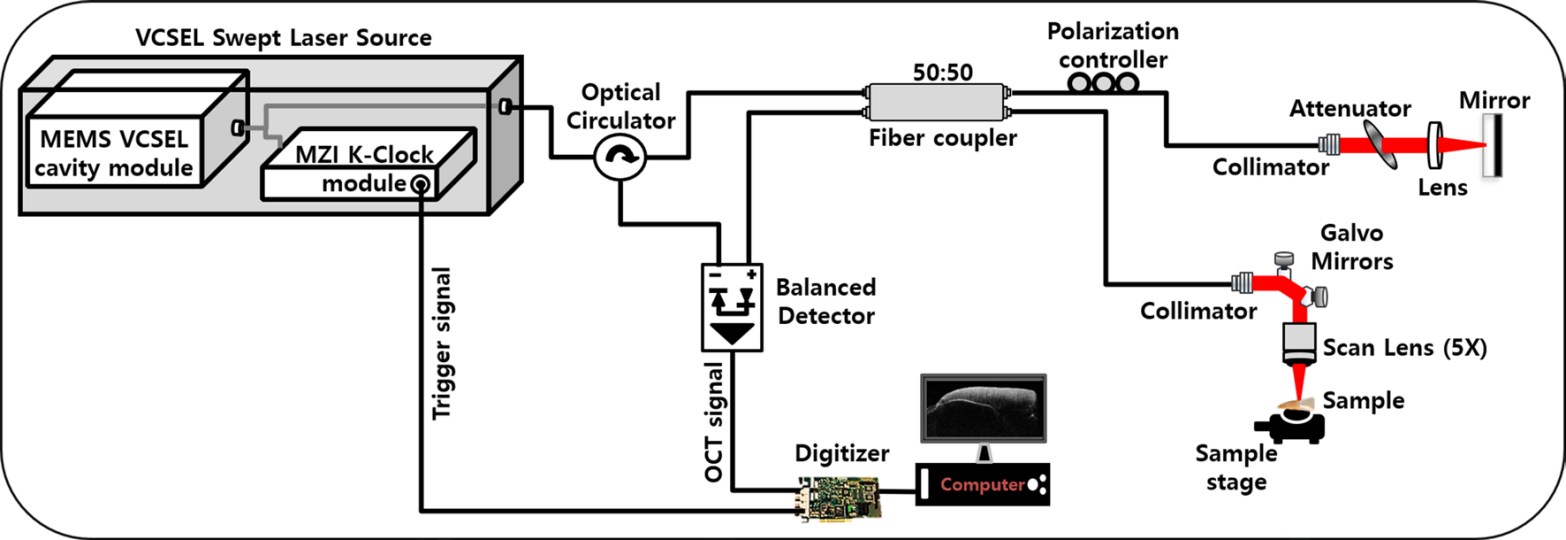
Schematic representation of the swept-source optical coherence tomography (SS-OCT) system.

A commercially available swept-source OCT (SS-OCT) system (OCS1310V1, THORLABS, New Jersey, USA) was used for imaging and measurements^30,31,43^. The graphical representation of the SS-OCT system is shown in Fig. 1. The SS-OCT system was powered by a microelectromechanical system (MEMS) tunable vertical-cavity surface emitting laser (VCSEL). The swept laser source was centered at 1310 nm, and the sweep rate was 100 kHz. The full-width at half maximum (FWHM) of the spectral bandwidth of the swept-source laser was 97 nm. A portion of the output beam from the VCSEL laser cavity module was connected to a Mach–Zehnder interferometer (MZI) clock module to obtain real-time optical clocking; using this, the OCT spectral fringes can be evenly sampled in wavenumbers with the referenced MZI signals, thereby eliminating the necessity of post processing for fringe resampling. The remaining beam of the source laser was coupled to the main OCT interferometer setup, which was connected via an optical circulator. The first output arm of the circulator was connected to a 50:50 optical coupler. The outlets of the optical coupler were directed to the sample and reference arm setups.

The interference signal (from the backscattered optical signals from the sample and reference arms) obtained from the output end of the coupler, along with the second output arm from the circulator arm, was connected to the positive and negative terminals of the dual balanced photodetector^43^. The sample arm setup comprised a 2D galvanometer scanner and an objective scan lens (LSM03, THORLABS, New Jersey, USA) of NA = 0.055. The reference arm setup comprised a collimator, lens, and highly reflective mirror. The interference signal from the dual balanced photodetector was linearly sampled by a 12-bit, 500 MS/s data acquisition card. Thus, the depth dependent reflectivity profile (A-line) was produced by fast Fourier transformation of the sampled fringe signals and by using the galvanometer scanner two-dimensional cross-sectional OCT image generated in real-tim. The sensitivity of the OCT system was 105 dB. The axial and transverse resolutions of the system (in air) were 18 and 25 *µ*m respectively^43^. The acquired B-scan (2D image) data after processing consisted of 2000 A-scans (depth scans) with each A-scan consisting of 1407 pixels. The three-dimensional image (C-scan) was formed by acquiring 500 successive B-scans. The axial and transverse resolutions measured in air were 16 and 25 *µ*m respectively. The scan range of one 2D cross-sectional image (B-scan) was set to 5 mm, and the volumetric scan range (C-scan) was set to 5×5 mm^43^.

### Depth intensity profile analysis algorithm

**Figure 2:**
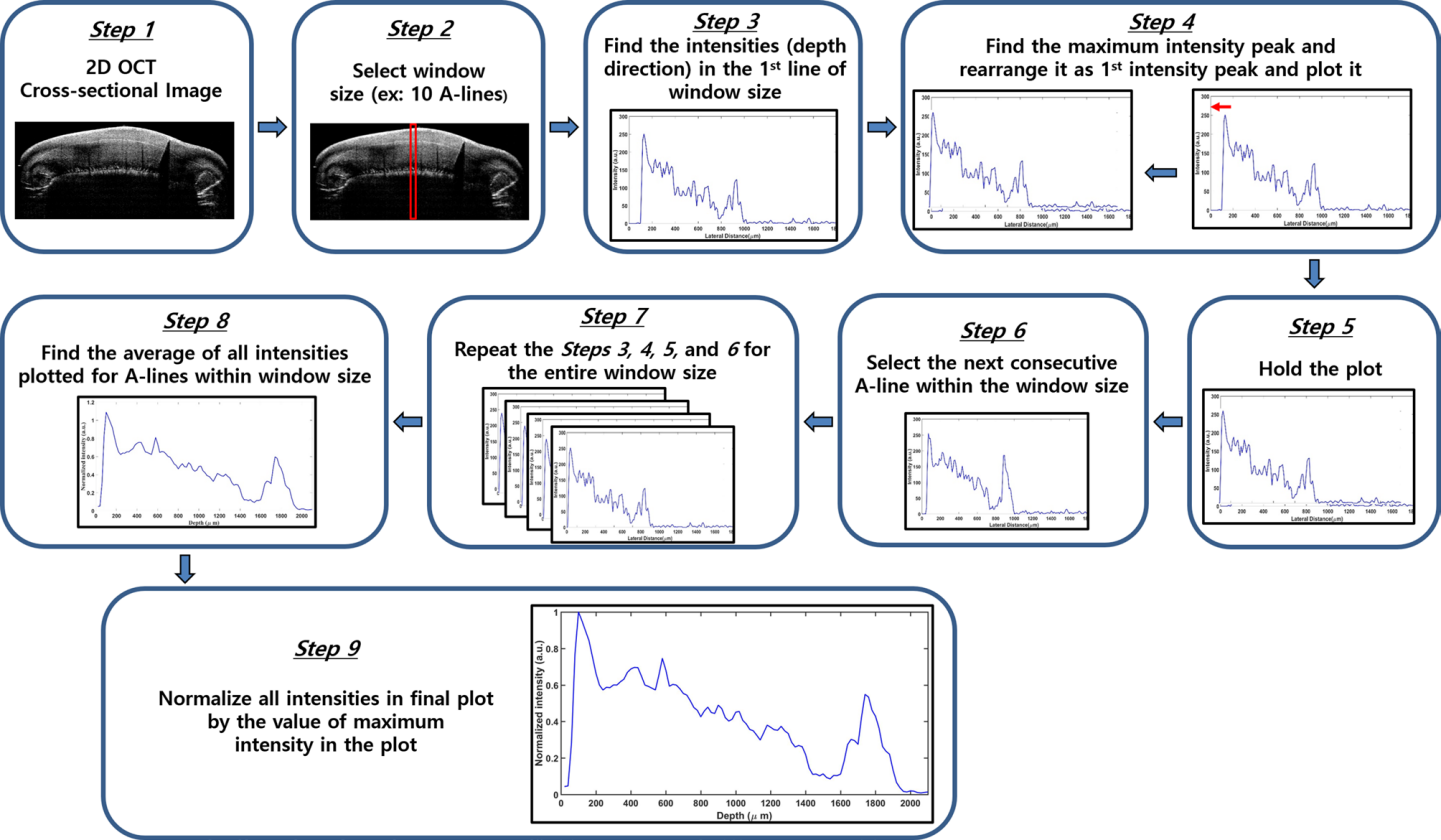
Flow diagram of depth intensity profile analysis algorithm.

To analyze the OCT images, a MATLAB-based software program was developed and used for intensity peak detection in the depth direction of the cross-sectional 2D OCT images^23,30,31,43^. The graphical representation of the flow diagram for the algorithm is shown in Fig. 2. The program searches for intensity peaks in the OCT images that are given within the desired window size. A window size of 10 was used for all the samples. Following this, the algorithm sequentially detected the maximum intensity of the A-scan (depth scan) signals. Then, the program rearranged all the detected peak positions of the A-scans within the window size while matching peak intensity indexes in the A-scans to flatten the image. The index positions with respect to high intensity are rearranged and matched linearly to obtain a flattened plot. The rearrangement was executed such that the first intensity peaks were retrieved from every A-scan of the 2D image and plotted at the beginning of the A-scan plot. It is noted that the absence of intensity peaks in the plot may be due to the presence of layers that are smaller than the maximum resolution of the system. Finally, all the rearranged and flattened A-scan lines were summed and averaged to obtain the averaged depth intensity profile. Then, the obtained depth intensity profiles were divided by the maximum values to obtain the normalized depth intensity plots for the desired window size of the 2D OCT image.

### Algorithm for analysis of enamel loss and abrasion by evaluating structural continuity

**Figure 3:**
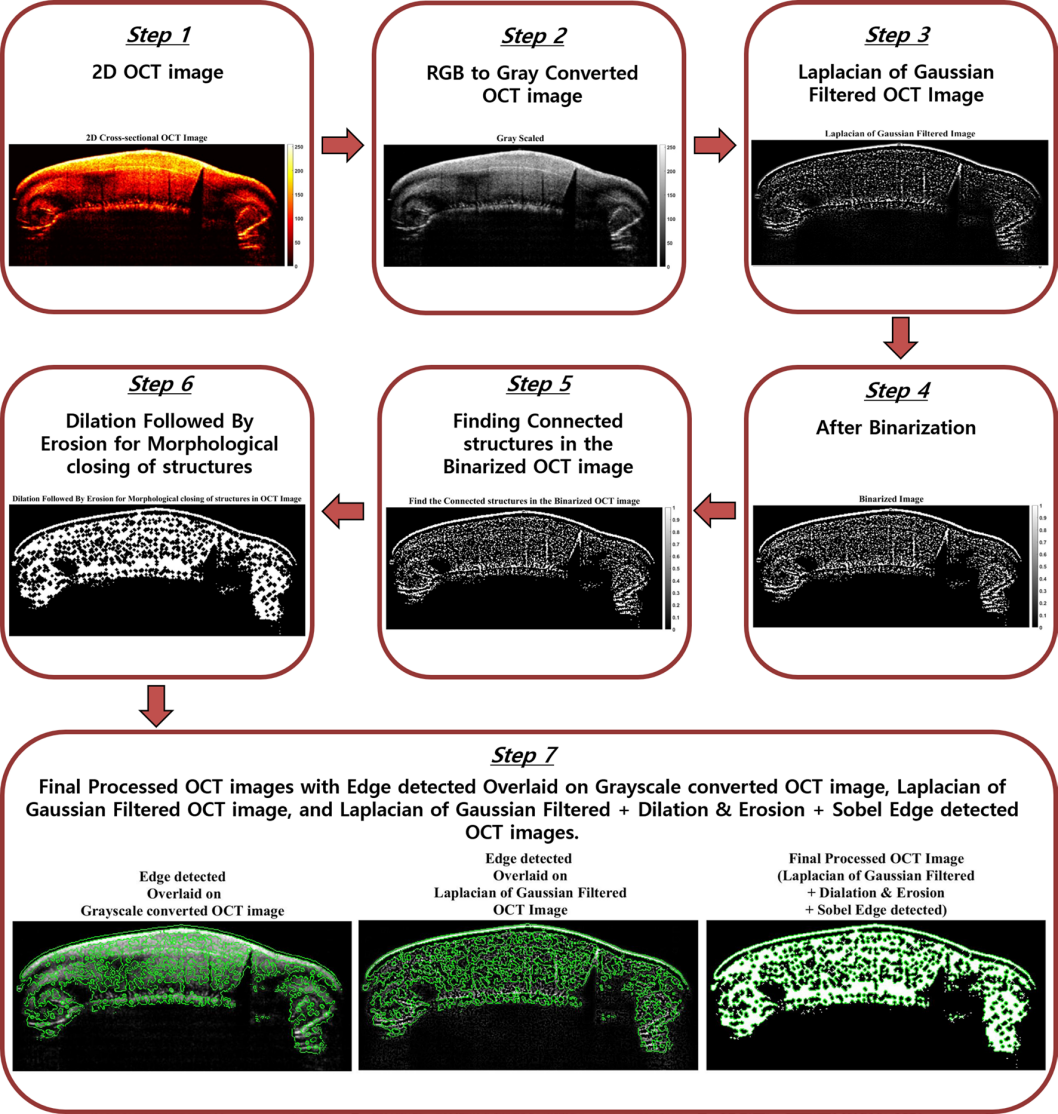
Stepwise representation of the algorithm for analyzing enamel loss and abrasion from an OCT image.

To analyze and assess the loss of enamel in the tooth and to also identify enamel abrasion on the tooth surface, a software algorithm was built on the MATLAB platform for assessing the structural connectivity. The flow diagram for the step-by-step process of the algorithm is shown in Fig. 3. The Execution of the program starts with the grayscale conversion of the obtained 2D cross-sectional OCT images (step 1 and step 2). This is followed by a rotationally symmetric Laplacian of Gaussian filtering of the OCT image (step 3) for detecting the edge and reducing noise to smoothen the image. Then, the edge-detected image was binarized (step 4) using a binarization operator.

Following this, the connected components (step 5) in the OCT images were traced with a default connectivity trace setting of 8. The image is then subject to morphological erosion for to remove unconnected structures and dilation to make the connected structures more visible (step 6). Sobel edge detection was then performed (step 7), and the edges detected by the Laplacian of Gaussian operation are overlaid on the grayscale converted cross-sectional OCT image (step 7, left image) and binarized OCT image (step 7, middle image). The final processed OCT image is the image that has undergone Laplacian of Gaussian filtering, dilation, erosion, and Sobel edge detection (step 7, right image). By introducing the morphological closing operation to the edge-detected image, it is possible to calculate the total enamel (intensities of connected structures) within the tooth; furthermore, by overlaying the final processed image on the binarized image, the growth by morphological closing of the tooth surface can be visualized. This morphological growth/protrusion correlates to the site of enamel abrasion on the tooth surface in the treated regions.

### Algorithm for intensity-based layer segmentation

**Figure 4:**
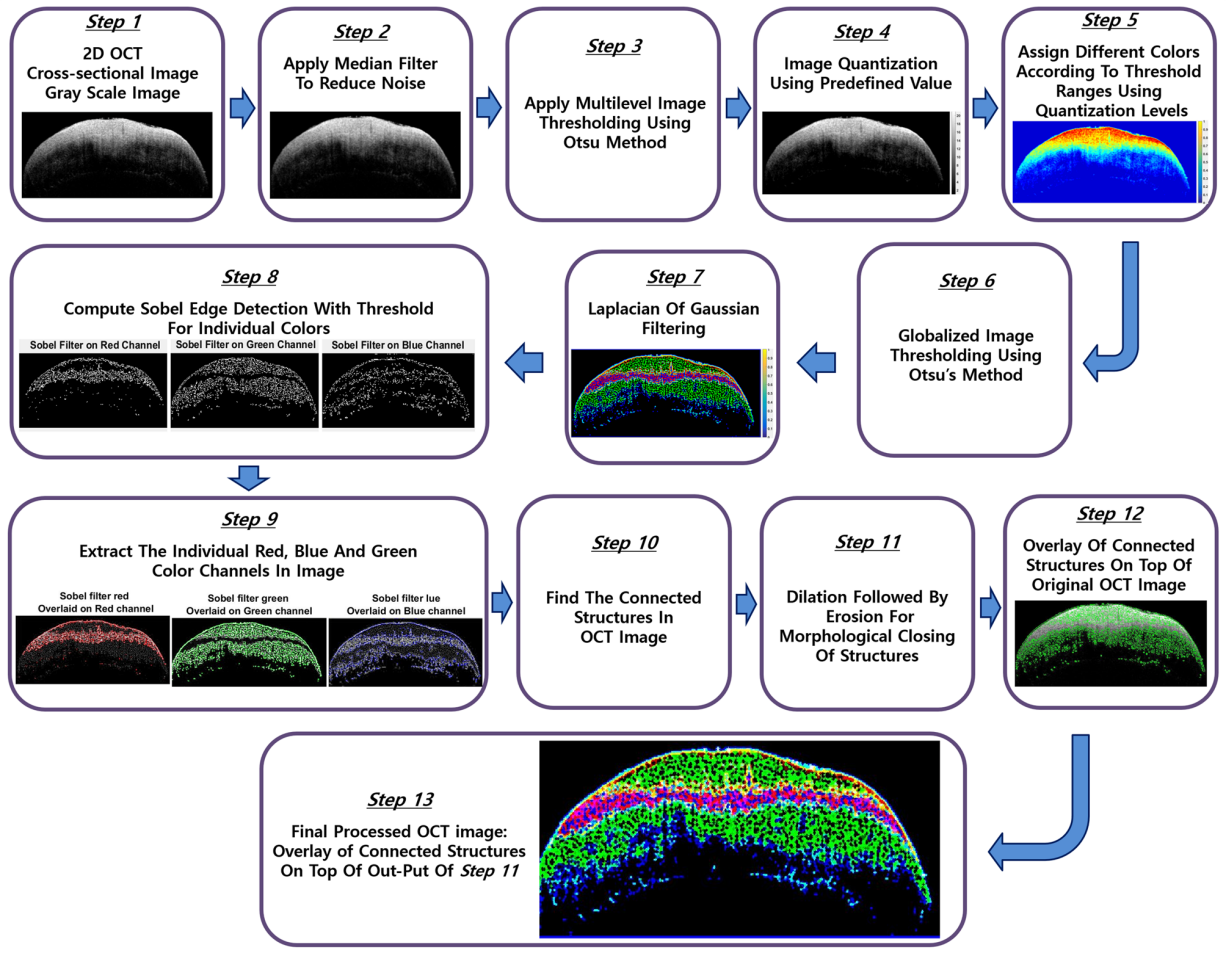
A step-by-step graphical explanation of the Algorithm for intensity-based layer segmentation method.

For further analysis of the internal enamel layer in OCT cross-section images, a layer segmentation algorithm based on the intensity of the internal enamel layer was built and compiled. The program was built using MATLAB. It is a well-known concept that the intensity changes in OCT images correspond to the variations of the refractive index of the sample morphology. Furthermore, the intensity fall-off in OCT images change/reduce as the depth increases. This is owing to the optical property of the light spectrum. To utilize this property of the OCT imaging system, an intensity-based layer segmentation analysis was proposed. A step-by-step graphical explanation of this algorithm is shown in Fig. 4. The built algorithm is initialized with the grayscale conversion of the 2D cross-sectional OCT image (step 1), and the converted image is then median filtered (step 2) to give a smoothed image with individual pixels being the median values of their 5-by-5 neighborhoods of the corresponding pixels in the original image.

The resultant image is a multilevel threshold (step 3) computed using Otsu’s method for setting the ranges of thresholding for the image. This is followed by image quantization (step 4) using the predefined threshold (same as the multithreshold value). With the multithreshold quantized values, the different ranges of intensities are assigned unique colors (step 5), followed by globalized image thresholding using Otsu’s method (step 6). Thereafter, the image is processed with a rotationally symmetric Laplacian of Gaussian filtering (step 7) for detecting the edge within each discrete RGB color in the image. This is followed by extracting the individual red, green, and blue color channels from the processed image, for which a Sobel filter edge detection is applied individually by the color channels with unique threshold values determined by trial and error for the best outcome (step 8). After the edges within the individual color ranges are detected, the individual red, green, and blue channels are extracted separately (step 9). In these extracted color channels, the connected components/structures are traced with a default connectivity trace setting of 8, and the individual channels are subjected to morphological closing operations, i.e., erosion for removal of unconnected structures and dilation for making the connected structures more visible (step 11). To view the connected structure and pattern in the OCT image, the original OCT image is overlapped with all the connected structures from the individual channels (step 12). As a final step, the morphologically closed structures of the individual colors are overlaid on the connected structures of the OCT image (step 13). From the final processed OCT image, it is seen that the internal enamel layers are segmented by intensity-based layer segmentation. This helps visualize the sublayer thickness variations in the OCT images.

### Ethics

All the experiments were approved and were carried out as per the guidelines of the Institutional Animal and Human Care and Use Committee of Kyungpook National University (No. 2017-0145-1). The collected tooth samples were extracted for orthodontic and periodontal reasons, which were sorted and stored anonymously without any retraceable or identifiable information of patients. Since our study did not involve any live human or live animal participation, the need for consent was waived by the ethics committee, which approved this study. Hence there was no reason to obtain an informed consent or personal data collection of any kind.

## Results and discussion

### Cross-sectional, *En face* and volumetric image analysis using OCT

**Figure 5:**
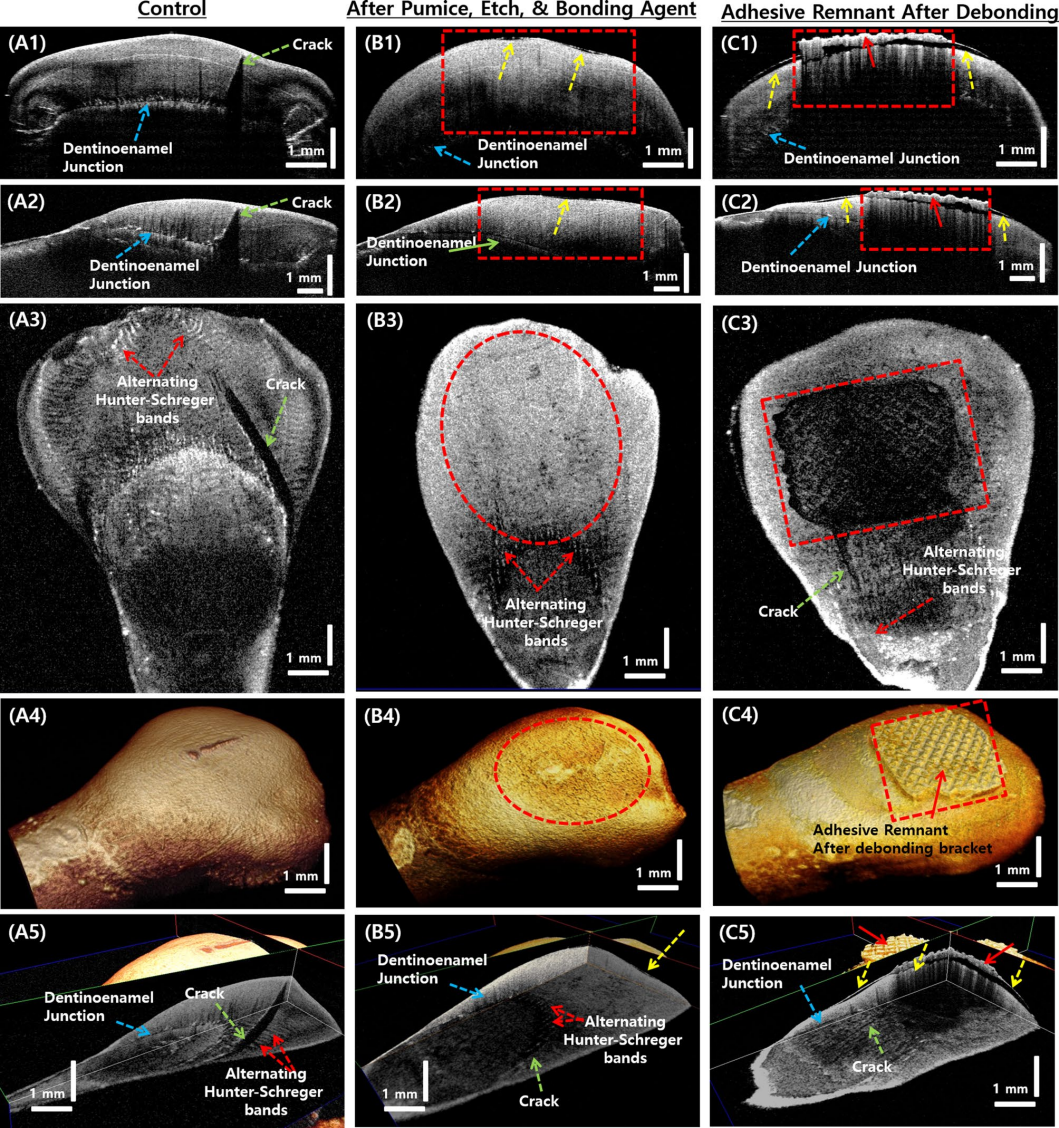
2D and 3D OCT images of tooth samples that underwent control, etch, and pumice followed by etching and bonding agent procedures. (**A1**–**A5**) are the control sample OCT images, (**B1**–**B5**) are the OCT images of a tooth sample that underwent pumice, etching and bonding agent application, and (**C1–C5**) are the 2D and 3D OCT images of a tooth sample after bracket debonding with the presence of adhesive remnant. Regions within the rectangular and circular dashed areas indicate the parts of the tooth surface that underwent the treatment procedures.

After each treatment process, all teeth samples were imaged using the SS-OCT system. The samples were scanned with the occlusal surface facing the scan lens. The 5 mm × 5 mm scan range was selected for volumetric scanning of the entire occlusal surface of the sample. Figure 5 shows the cross-sectional, *en face*, and 3D volumetric OCT images of the samples in control group (Fig. 5, A1–A5), after the pumice followed by etching, and application of bonding agent (Fig. 5, B1–B5) to the tooth surface, and images of the tooth sample after bracket debonding with in presence of adhesive remnant (Fig. 5, C1–C5). In Fig. 5, A1 and A2 show the 2D OCT images of the control sample in the axial and sagittal planes, and A3 is the *en face* image taken 500 *µ*m below the external tooth surface. A4 shows the 3D volumetric image of a control sample tooth. In the 2D and 3D images, the enamel, dentinoenamel junction, and alternating Hunter–Schreger bands within enamel structures are clearly visible. A5 shows the volumetric OCT image with all three sectional planes showing the internal morphology of the tooth sample. Images B1-B5 are tooth samples that underwent pumice grinding followed by etching and application of bonding agent to the tooth surface. In Fig. 5, B1–B5 are the images of a tooth sample that underwent pumice, etching, and bonding agent application. In Fig. 5, B1 and B2 show the 2D OCT images of the tooth sample in the axial and sagittal planes; B3 is the *en face* image taken 500 *µ*m below the external tooth surface; B4 shows the 3D volumetric representation of the sample; B5 shows the volumetric OCT image with all three sectional planes showing the internal morphology of the treated tooth sample. Figure 5 shows the 2D and 3D OCT images of a tooth sample after bracket debonding (removal) and before removal of adhesive remnant after debonding. C1 is the 2D OCT image in the axial plane, and C2 is the 2D OCT image in the sagittal plane. C3 is the *en face* OCT image 500 *µ*m below the tooth surface; C4 and C5 are the 3D volumetric OCT image and the volumetric OCT image with all three image planes respectively. The red dashed box regions highlight the adhesive remnants; yellow dashed arrows indicate enamel abrasion on the tooth surface due to bracket removal and pre-treatment procedures. Owing to the reflective nature of the adhesive compound used, the internal enamel structures are not clearly visible. The dashed green arrows in the OCT images indicate the naturally occurring micro-cracks in the tooth, and the dashed yellow arrows indicate the enamel abrasions on the tooth surface.

**Figure 6:**
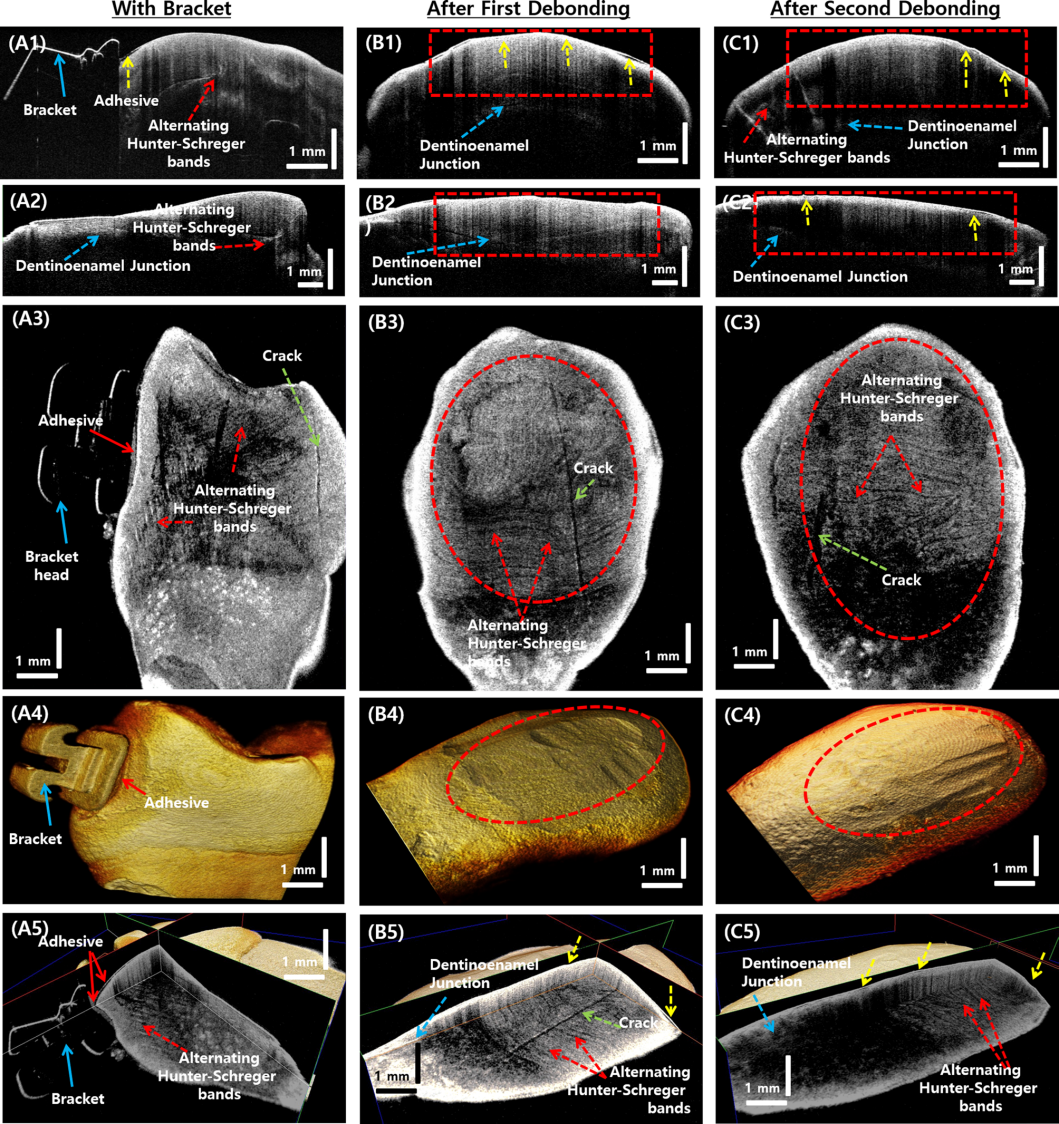
2D and 3D OCT images of tooth samples after bracket bonding, firstdebonding of the bracket, and second debonding of the bracket. (**A1–A5**) are the OCT images of a tooth sample after bracket bonding, (**B1**–**B5**) are the OCT images of a tooth sample after the first debonding of the bracket, and (**C1–C5**) are the OCT images of a tooth sample after second debonding from a tooth sample. Regions within the rectangular and circular dashed areas indicate the parts of the tooth surface that underwent the treatment procedures.

Figure 6, A1–A5 are the 2D, 3D, and *en face* OCT images of the tooth sample, which are firmly fixed with an orthodontic bracket. In Fig. 6, A1 and A2 show the 2D OCT images of the axial and sagittal planes respectively; A3 is the *en face* image taken 500 *µ*m below the external tooth surface; A4 and A5 show the 3D volumetric image and volumetric OCT image with all three sectional planes showing the internal morphology of the sample. The blue solid arrow indicates the orthodontic bracket, and the solid red arrow shows the adhesive remnant below the bracket. Figure 6, B1–B5 are the 2D, 3D, and *en face* OCT images of the tooth sample after the first removal (debonding) of the bracket. Cross-sectional images in the axial and sagittal planes are shown in B1 and B2. The *en face* image 500 *µ*m below the external tooth surface is shown in B3, and B4 and B5 are the 3D volumetric image and the volumetric OCT image with all three sectional planes showing the internal morphology of the sample. It is seen that the occurrence of enamel abrasion increased considerably, which is shown using dashed yellow arrows. This could be due to the stress induced on the tooth surface during debonding and during the adhesive remnant cleaning process. Similarly, in Fig. 6 C1–C5, represents the removal of the bracket from the tooth after two attachments and removals, the overall enamel content and enamel abrasion is further increased. This can be observed in the cross-sectional images in the axial and sagittal planes as shown in C1 and C2, in the *en face* image 500 *µ*m below the external tooth surface as shown in B3, and also in B4 and B5, which are the 3D volumetric image and the volumetric OCT image with all three sectional planes showing the internal morphology of the sample. It can also be seen that the visibility of the dentinoenamel junction is reduced in the sample after debonding and adhesive cleaning.

### Depth intensity profile analysis of cross-sectional OCT images

**Figure 7:**
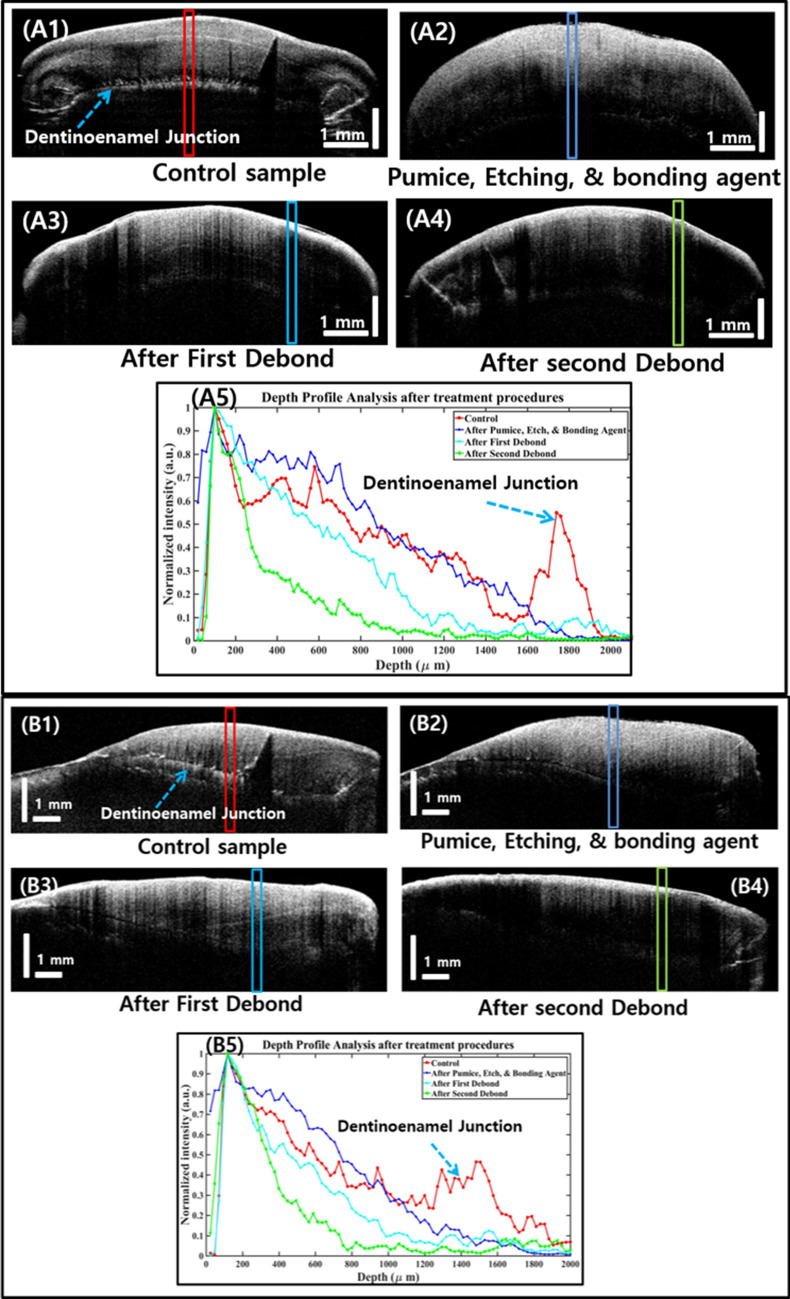
Depth intensity profile analysis of cross-sectional OCT images of the tooth sample after each stage of treatment procedures. (**A1**–**A4**) and (**B1–B4**) are the cross-sectional OCT images in the axial plane and sagittal plane, respectively. Tooth samples that are taken as control (**A1**, **B1**), after pumice, etching, and bonding agent step (**A2**, **B2**), after first debonding and adhesive remnant cleaning (**A3**, **B3**), and after second debonding followed by adhesive remnant cleaning procedure (**A4**, **B4**). (**A5**) and (**B5**) are the depth intensity profile plots of OCT images, respectively. Solid rectangular boxes (red, black, light blue, dark blue, and green) are the regions within which the depth profile analyses are executed.

The best representative cross-sectional OCT images of all 30 teeth samples underwent the depth intensity profile analysis for enamel layer detection. As mentioned in section II (C), the algorithm is used for depth intensity profile analysis of teeth samples after treatment. Representative OCT images, along with their plots, are shown in Fig. 7. In Fig. 7, A1–A4 and B1–B4 are the cross-sectional OCT images in the axial and sagittal planes, respectively. Tooth samples are taken as control (A1, B1), after pumice, etching, and bonding agent step (A2, B2), after first debonding and adhesive remnant cleaning (A3, B3), and after second debonding followed by adhesive remnant cleaning (A4, B4). A5 and B5 are the depth intensity profile plots of the OCT images. The solid rectangular boxes (red, black, light blue, dark blue, and green) are the regions within which the depth intensity profile analyses are executed. The window size is set to 10 within the selected rectangular box regions. From the obtained plots, it is seen that the enamel thickness decreases after each step of the treatment procedure. It is noted here that the maximum reduction in enamel thickness is observed in the group with the second debonding of the bracket followed by adhesive remnant cleaning.

The second highest enamel thickness and enamel intensity were measured in the control group. Further, the second-highest peak represents the dentinoenamel junction. The highest intensity and enamel thickness were measured in the tooth samples that underwent pumice, etching followed by application of bonding agent, and this is due to the additional microstructure of the bonding material that is combined with the enamel surface. Additionally, there is a considerable amount of intensity reduction of enamel in the second debonded group as compared to the first debonded group. This supports the understanding of enamel loss and abrasion caused during the bonding and debonding processes. The stated results are to be interpreted while considering the fact that, usually in clinical orthodontic practices, during bracket bonding procedures, the treatment process of pumice, etching and application of bonding agent is seen as an interlinked successive process. This is taken into consideration in the present study. Moisture contamination of the enamel surface during the bracket placement procedures can lead to bond failures during the orthodontic treatment^44^. Oral contaminants (saliva, blood, gingival fluid, water) reduce the bond strength significantly, and this is considered to be the most common reason for bond failure with composite resin^45^. Enamel surface contamination during bonding procedures can occur mainly at two critical times. First is after the surface has been etched and second after the primer has been applied before placement of the bracket^46^. Contamination before priming would inevitably cause the formation of a smear layer. This layer, consisting mainly of proteins, covers the etched surface within seconds. Most of the porosities become plugged, and the penetration of the resin is impaired, which results in resin tags of insufficient number and length. Hence, it is vital to prevent any kind of moisture, that the area be kept clean and dry and the bonding agent applied immediately followed by bracket placement. This procedure also exactly replicates the clinical scenario and thus is emulated in our present study.

### Evaluation of structural connectivity in tooth images to analyze enamel loss and enamel abrasion

**Figure 8:**
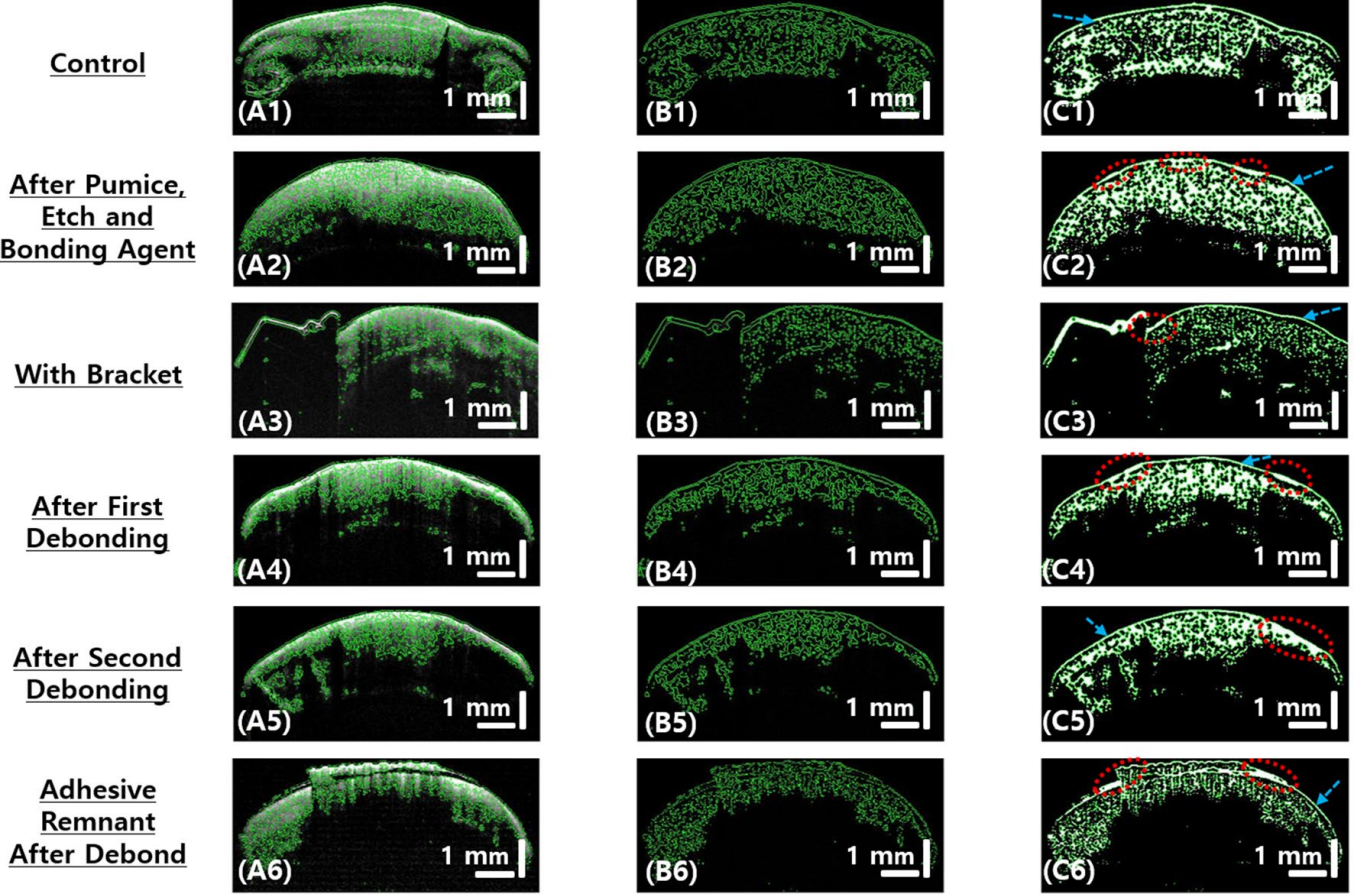
Structural connectivity for analyzing the enamel loss and enamel abrasion on the tooth surface. (**A1–A6**) is the cross-sectional OCT images after each step overlaid with its respective connected structures. (**B1**–**B6**) are the connected structures in enamel. (**C1–C6**) are the morphological closing for connected structures in the OCT images obtained after each treatment procedure. Dashed red circles indicate the enamel abrasion due to the treatment procedure. Blue dashed arrow shows the thin layer of the outer enamel boundary.

Using the methodology previously explained in section II (D), the evaluation of structural connectivity in tooth structures was carried out on the selected cross-sectional OCT images that were obtained for all 30 samples after each treatment procedure. The post-processed representative tooth images are shown in Fig. 8. In Fig. 8, A1–A6 are the original grayscale cross-sectional OCT images obtained in the axial plane, which are overlaid with the connected structures obtained using the structural connectivity evaluation algorithm. Fig. 8, B1–B6 are the connected structures portraying the connectivity in the enamel layers within the tooth samples. Fig. 8, C1–C6 are the morphological closing operations performed on the structural connectivity of the enamel layer. By performing the morphological closing operation on the obtained connected structures, the total amount of enamel present in the tooth samples can be determined. Thus, from the obtained processed images, the continuity and enamel reduction with the treatment process are confirmed.

The thin layer of outer enamel boundary (blue dashed arrow) can be distinctly seen in the processed OCT images, which is not visible in the original cross-sectional OCT images. Furthermore, the final processed images show the continuity of enamel structures, which can be seen as continuous white intensities. The loss of enamel, i.e., enamel abrasion on the tooth surface, can be seen as white intensities on the thin enamel outer boundary, as highlighted in C2-C6 by dashed red circles. Further, in the processed OCT image taken after the second debonding followed by adhesive remnant cleaning (C5), it can be clearly seen as the merging of the thin outer enamel boundary with inner structures (shown within red dashed circle). This could be a result of the multiple etching, pumice, and cleaning processes of adhesive remnant layers.

### Intensity-based layer segmentation analysis for internal enamel layer visualization

**Figure 9:**
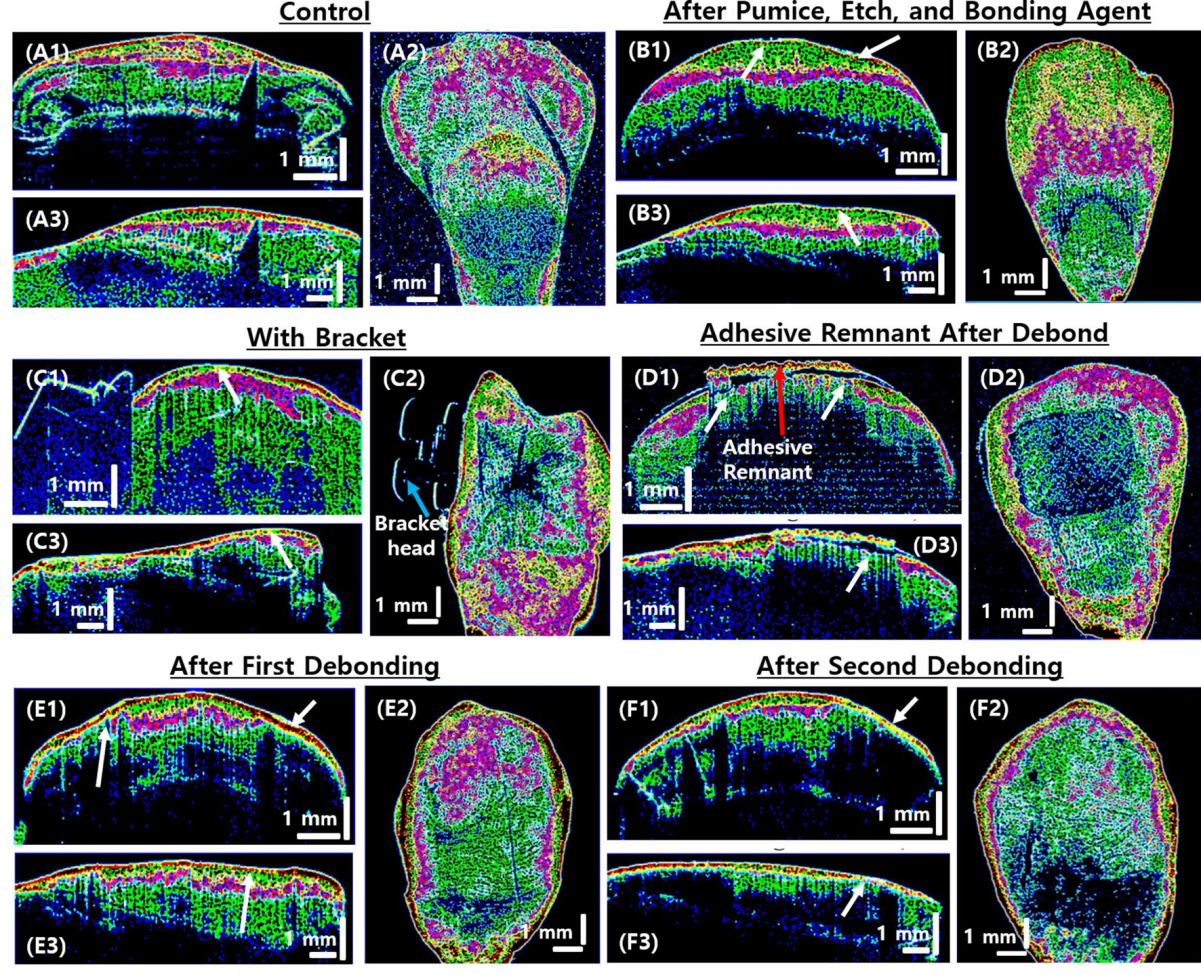
Layer segmentation analysis for internal enamel layer visualization in cross-sectional OCT images.2D cross-sectional OCT image in axial plane (**A1**, **B1**, **C1**, **D1**, **E1**, and **F1**) and sagittal plane (**A3**, **B3**, **C3**, **D3**, **E3**, and **F3**), and *en face* OCT images (**A2**, **B2**, **C2**, **D2**, **E2**, and **F2**) at 500 µm below the tooth surface. White arrows indicate enamel abrasion caused by treatment procedures.

Figure 9 shows the intensity-based layer segmented OCT images. By utilizing the methodology explained in section II (E), the selected cross-sectional OCT images (after each treatment procedures) in axial plane (A1, B1, C1, D1, E1, and F1) and sagittal plane (A3, B3, C3, D3, E3, and F3), and *en face* OCT images (A2, B2, C2, D2, E2, and F2) that are obtained 500 *µ*m below the tooth surface are post processed for internal enamel layer segmentation analysis. OCT images of Fig. 9 (A1-A3) belong to the control group, Fig. 9 (B1-B3) are representative images of a tooth sample treated with pumice, etch and application of bonding agent, Fig. 9 (C1-C3) are OCT images of a representative tooth sample with bracket firmly attached, Fig. 9 (D1-D3) are images of a tooth sample that is obtained before the cleaning of adhesive remnant on tooth surface, and Fig. 9 (E1–E3) are the images obtained after first debonding of bracket followed by cleaning of adhesive remnant; similarly, Fig. 9 (F1–F3) are OCT images obtained from a tooth sample that underwent bonding twice, debonding, and remnant cleaning processes.

As explained in the methodology, in each of the OCT images, the layers are segmented using different colors depending on the defined intensity ranges. Furthermore, by finding the connected structures within the enamel layer and with the help of morphological closing operation, it is possible to see the continuity in the intensity ranges. This allowed visualizing the internal enamel structures with more clarity when compared to conventional grayscale OCT. The white arrows in the images indicate the enamel abrasion caused by different treatment procedures. It can be observed that the internal layers in magenta colored and red-colored are reduced in each treatment process with respect to the amount of enamel damage that occurred during the treatment process. In images F1-F3 that were obtained after bracket bonding and debonding is executed twice, the internal red and magenta colored structures are almost diminished (the region around white arrow). Moreover, we could see the additional accumulation of green segmented layers in the pumice, etch and bonding agent applied tooth sample (B1-B3), which could be due to the infusion of microstructures by a bonding agent. The green segmented layers that constitute most of the enamel structures appear to be visibly reduced in the successive treatment processes.

Using the *en face* images obtained from the intensity-based layer segmented analysis of all tooth samples (the representative images for each treatment group is shown in Fig. 9 A2, B2, E2, and F2), the total enamel present in the tooth samples at 500 *µ*m below the tooth surface is calculated. The total enamel present in these images corresponds to the total pixel intensities in continuity in the *en face* images. The calculated values for enamel present for each treatment group, i.e., the average (mean), the minimum, and the maximum values along with standard deviation, is given in Table 1.

**Figure 10:**
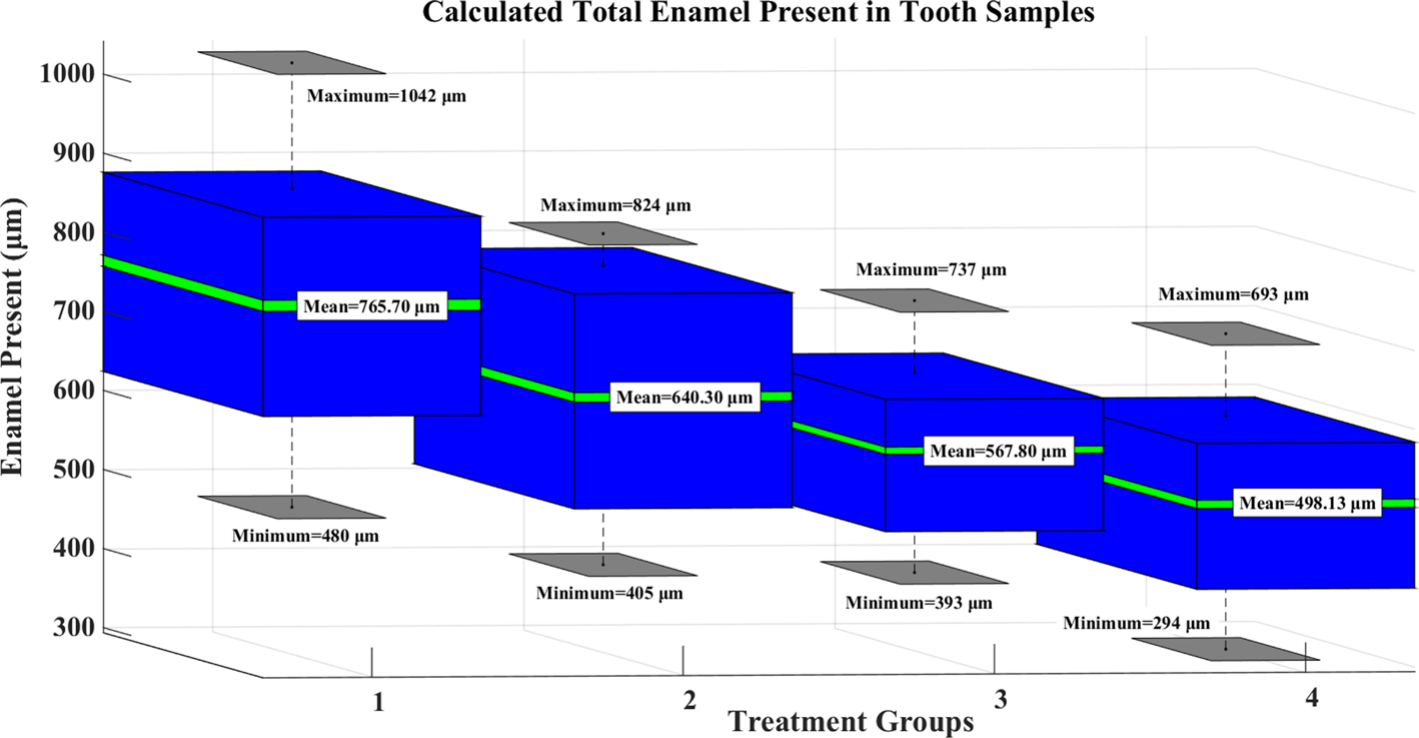
Total calculated enamel present in the *en face* OCT images of tooth samples after each treatment procedure. Treatment groups 1 to 4 represent the control group; after pumice, etch and bonding agent application group; after first debonded sample group; and after second debonding sample group. The mean, maximum, and minimum values in each group are given in the 3D box plot.

For an easier representation of the calculated total enamel in the *en face* images of all tooth samples, Fig. 10 shows the 3D box plot representing the mean, maximum, and minimum of all treatment groups. In Fig. 10, the X-axis is represented with treatment groups from 1-4. The treatment groups in order from 1-4, control (group 1), after pumice, etch, and bonding agent applied (group 2), after first debonding (group 3), and after second debonding (group 4) shows the clear reduction in enamel content in the tooth samples after each treatment. The mean enamel presence in group 1 is 765.70 µm with a minimum value of 480 µm and a maximum of 1042 µm. A reduction in the enamel of 125.40 µm (mean difference) was observed after pumice, etch, and bonding agent application.

It should be noted that the applied bonding agent constitutes an increase in the overall content of the tooth after treatment. In general, as the OCT images cannot differentiate between two materials by their chemical compositions, the overall calculated pixels in group 2 samples comprise enamel presence and the applied bonding agent. Furthermore, the total average reduction of enamel content in group 4 samples compared to group 3 samples is 69.67 *µ*m. The total enamel reduction after all treatment procedures is about 267.57 *µ*m (the mean difference between group 1 and group 4). Additionally, it was observed that after the pumice, etching and bonding agent treatment an overall enamel loss of 115.40 µm can be seen. This may be due to the pumice pre-bonding procedure by itself, as it is a procedure that can cause enamel loss to a minimal degree and this value has to be taken into account in the total enamel loss as seen after the end of treatment. In clinical orthodontic practices, the initial step in bonding an orthodontic bracket is to thoroughly clean the enamel surface and to attain maximum surface energy for a good bond strength of the bracket. This is usually done with use of pumice^47^. This pretreatment removes any organic material including acquired pellicle, any pre-existing stains present on the teeth surface and also to increases the surface roughness of the tooth to make it more susceptible for acid etching. Furthermore, it is worthy to be noted that this step of use of pumice, has no effect on bond failure rates^48^. Hence, to achieve maximum clinical treatment scenario, the enamel surface of all tooth samples were polished for 10 seconds using pumice. This in-turn can cause abrasion on the enamel surface. The loss of enamel is usually miniscule. The use of pumice is a widely accepted routing process in various treatments used in clinical orthodontics and in other fields of dentistry. All the samples have been subjected to the same procedure, removing any bias. The calculated enamel in each tooth sample after every treatment procedure is given in the supplementary Table S1.

For further analysis, the obtained results were statistically analyzed by repeated measures ANOVA and intergroup comparisons. All the statistical analysis was performed using SPSS version 18. A p-value of <0.05 was considered statistically significant. The normality of the data was checked by the Kolmogorov–Smirnov test. A comparison of the mean values was made using repeated measures ANOVA with post-hoc Bonferroni test. A comparison of the mean values from the baseline (control group) through second debonding showed significant differences in the mean values (Table 1). As given in in Table 2, the post-hoc analysis showed that comparisons between Baseline and After First Debonding, Baseline and After Second Debonding, and After Pumice, Etch, & Bonding Agent and After Second Debonding showed significant differences. No other significant differences were observed.

**Table 1:** Repeated measures ANOVA with post-hoc BONFERRONI test.

	Mean (μm)	Standard deviation (μm)	*P*-value
Baseline (Control)	765.70	168.00	< 0.001; Sig
After Pumice, Etch, & Bonding Agent	640.30	140.71	
After First Debonding	567.80	106.56	
After Second Debonding	498.13	117.50	

Sig: Significant; NS: Not Significant.

**Table 2:** Intergroup comparison for enamel.

Inter-group comparisons	*P *Value
Baseline vs After Pumice, Etch, & Bonding Agent	0.055; Sig
Baseline vs After First Debonding	< 0.001; Sig
Baseline vs After Second Debonding	< 0.001; Sig
After Pumice, Etch, & Bonding Agent vs After First Debonding	0.282; NS
After Pumice, Etch, & Bonding Agent vs After Second Debonding	0.002; Sig
After First Debonding vs After Second Debonding	0.067; NS

Sig: Significant; NS: Not Significant.

## Conclusion

In this study, we have shown the practical applicability of optical coherence tomography (OCT) as a non-ionizing and nondestructive assessment tool for measuring enamel loss and enamel abrasions caused by different treatment processes during orthodontic bracket bonding and debonding processes. The two-dimensional and volumetric imaging capabilities of the OCT system is used for initial assessment of enamel presence after the individual treatment process. By implementing the depth intensity profile analysis for 2D OCT images, we calculated the changes in the individual internal layer thickness and enamel intensity variations after each treatment process, thus showing that the highest intensity and enamel thickness are observed in the tooth samples that underwent pumice etching followed by application of the bonding agent; these characteristics could also be attributed to the additional microstructures of the bonding material that may have been combined with the enamel surface. Further, it was observed that there was a considerable amount of intensity reduction of the enamel in the group that was debonded for the second time as compared to that debonded the first time; this supports the understanding of enamel loss and abrasion caused during the bonding and debonding processes.

For further analysis of the enamel layers, the structural connectivity in the tooth structures are evaluated from select cross-sectional OCT images from all samples after each treatment process. The post processed OCT images show the continuity of enamel structures, which can be seen as continuous white intensities. The loss of enamel, i.e., enamel abrasion on the tooth surface, can also be seen as a continuity of white intensities on the thin enamel outer boundary, which merged with successive internal enamel structures. Furthermore, for better visualization of the internal enamel layers, intensity-based layer segmentation was carried out on 2D and *en face* OCT images as compared to conventional grayscale OCT images. Additionally, by finding the connected structures within the enamel layer and with the help of the morphological closing operation, it was possible to see the continuity in the intensity ranges. This helped validate the findings that the internal enamel layers are reduced in each treatment process with respect to the amount of enamel damage occurring during the treatment processes; moreover, the internal layer immediately below the outer thin enamel boundary layer is almost diminished in the sample group where the bonding and debonding are performed twice at the same spot on the tooth surface. In recent years, the use of OCT has seen a rapid growth as a reliable non-ionizing and high resolution imaging modality for in vivo and in vitro in dental related experimental research and in clinincal trails^32,49^. Through this study, we have demonstrated the applicability of OCT as a diagnostic and an assessment tool for enamel loss and enamel abrasions on the tooth surface during orthodontic bracket bonding and debonding processes. Additionally, the utilization of the proposed post processing method for OCT images is expected to be of assistance to researchers and clinical practitioners for assessing the conditions of enamel structures during treatments and experiments.

## Supplementary Information

Supplementary Information.
