# Supplementary material for: Micron-scale human enamel layer characterization after orthodontic bracket debonding by intensity-based layer segmentation in optical coherence tomography images
Source: Sci Rep. 2021 May 25;11:10831. doi: 10.1038/s41598-021-90354-9 (PMC8149424; doi:10.1038/s41598-021-90354-9)
Supplement: Supplementary file 1 — Supplementary Information. [file 41598_2021_90354_MOESM1_ESM.docx]

**Micron-Scale Human Enamel Layer Characterization After Orthodontic Bracket Debonding by Intensity-Based Layer Segmentation in Optical Coherence Tomography Images**

Naresh Kumar Ravichandran1*,*2, Hemanth Tumkur Lakshmikantha3, Hyo-sang Park3, Mansik Jeon1*,∗* & Jeehyun Kim1

1*School of Electronics Engineering, College of IT Engineering, Kyungpook National University, 80 Daehakro, 41566, Bukgu, Daegu, Republic of Korea*

2*Center for Scientific Instrumentation, Korea Basic Science Institute, 169148 Gwahakro Yuseonggu, 34133, Daejeon, Republic of Korea*

3*Department of Orthodontics, School of Dentistry, Kyungpook National University, 41940, Daegu, Republic of Korea*

*****Correspondence: [msjeon@knu.ac.kr](mailto:msjeon@knu.ac.kr)

**Supporting Information:**

**Legend of supplementary table S1:**

**Supplementary Table S1:** The calculated total enamel present in individual tooth samples after every treatment procedure.

The total average reduction of enamel content in group 4 (after second debonding) samples compared to group 3 (after first debonding) samples is 69.67 *µ*m. The total enamel reduction after all treatment procedures is about 267.57 *µ*m (the mean difference between group 1 and group 4).

| **Calculated Total Enamel Present (μm) for Individual Samples** | | | | |
| --- | --- | --- | --- | --- |
| **Sample No.** | **Control** | **After Pumice, Etch, & Bonding Agent** | **After First Debonding** | **After Second Debonding** |
| 1 | 930 | 613 | 549 | 468 |
| 2 | 653 | 783 | 592 | 545 |
| 3 | 676 | 792 | 435 | 589 |
| 4 | 728 | 420 | 512 | 382 |
| 5 | 872 | 540 | 539 | 580 |
| 6 | 528 | 491 | 671 | 469 |
| 7 | 558 | 793 | 561 | 294 |
| 8 | 874 | 813 | 547 | 351 |
| 9 | 695 | 752 | 399 | 536 |
| 10 | 883 | 405 | 393 | 473 |
| 11 | 966 | 818 | 498 | 693 |
| 12 | 750 | 635 | 626 | 442 |
| 13 | 1003 | 475 | 708 | 595 |
| 14 | 971 | 796 | 651 | 646 |
| 15 | 1042 | 505 | 559 | 407 |
| 16 | 786 | 670 | 737 | 538 |
| 17 | 601 | 468 | 711 | 668 |
| 18 | 880 | 657 | 625 | 631 |
| 19 | 907 | 470 | 609 | 367 |
| 20 | 869 | 469 | 642 | 673 |
| 21 | 730 | 508 | 604 | 590 |
| 22 | 549 | 774 | 646 | 582 |
| 23 | 1037 | 544 | 437 | 553 |
| 24 | 555 | 824 | 456 | 294 |
| 25 | 833 | 596 | 725 | 416 |
| 26 | 480 | 614 | 474 | 399 |
| 27 | 624 | 740 | 712 | 477 |
| 28 | 702 | 758 | 596 | 358 |
| 29 | 511 | 667 | 402 | 348 |
| 30 | 778 | 819 | 418 | 580 |
| **Average** | **765.70** | **640.30** | **567.80** | **498.13** |
| **Standard**  **deviation** | **168.00** | **140.71** | **106.56** | **117.50** |
